# Matching Network Design for Ultrasonic Guided Wave Interdigital Transducers

**DOI:** 10.3390/s25175401

**Published:** 2025-09-01

**Authors:** Lorenzo Capineri

**Affiliations:** Dipartimento Ingegneria dell’Informazione, Università degli Studi di Firenze, 50139 Firenze, Italy; lorenzo.capineri@unifi.it

**Keywords:** interdigital transducer, matching network, ultrasonic guided wave, equivalent electric impedance, integrated driving electronic, network integration, structural health monitoring

## Abstract

**Highlights:**

This is a study of the analysis and design rules of a resonant matching network for ultrasonic guided wave interdigital transducers involving integrated implementation with a piezopolymer interdigital transducer and validation with low-voltage-power-supply-driving electronics.

**What are the main findings?**

- A passive component matching network for interdigital guided wave transducers;
- Transfer function analysis and design rules/methods.

**What are the implications of the main findings?**

- Easier integration of interdigital transducers into structural health monitoring systems;
- Driving electronic circuit with low power supply voltage.

**Abstract:**

Ultrasonic guided wave interdigital transducers realized with piezoelectric materials are of interest for structural health monitoring systems because of their capability of performing Lamb wave mode selection with respect to single-element transducers. Besides this advantage, the coverage of large areas with a minimum number of elements is an important challenge and the problem of efficient excitation with integrated electronics must be solved. This work proposes an electrical matching network topology made of L and C passive components that can be designed for the trade-off between electrical to mechanical conversion efficiency and bandwidth. The network circuit is analyzed considering the equivalent transducer impedance and the output impedance of the driving electronics. The design rules derived by the transfer function analysis are described and a case study for a piezopolymer IDT is presented. Finally, with the implementation of the integrated matching network with the connector of the IDT, the effect of cable capacitance is minimized. In conclusion this article is a contribution to the study of using IDT efficiently and in a versatile mode for different electronic front-ends that usually operate at low power supply voltage.

## 1. Introduction

Ultrasonic guided wave interdigitated transducers (UGWIDTs) are increasingly used in SHM systems [1,2,3] for the monitoring of both composite [4,5] and metallic [6] laminar structures, due to their ability to select propagation modes useful for detecting and monitoring mm- and sub-mm sized defects.

To cover large areas with a limited number of transducers, it is necessary to be able to shape the beam while simultaneously ensuring high transmitted power to ensure a good signal-to-noise ratio [7,8]. To maximize the power, the UGWIDT must be well matched with the driving electronics.

UGWIDTs are piezoelectric devices designed to operate at defined frequencies with a narrow bandwidth (typically tens of kHz). Typically, SHM systems are designed with operating frequencies selected in the 100 kHz–1 MHz range based on the characteristics of the laminate and therefore the dispersion curves.

Fabricating UGWIDTs with thin (20 μm–200 μm) piezoelectric materials (piezopolymer films [5], spray deposition [9], piezocomposites [10]) in the above frequency range results in a non-resonant piezoelectric device, meaning its equivalent electrical impedance is well approximated by an ohmic-capacitive impedance $Z_{L}={\;R}_{L}\;+\;1/{j\omega C}_{L}$. The resistive part of the impedance $R_{L}\;$ represents the conversion of electrical excitation power into mechanical power and also includes the piezoelectric material losses; the capacitance $C_{L}$ depends on the dielectric constant of the piezoelectric material, the thickness and the area of the electrode design and is frequency-dependent.

The objective of this work is to analyze a modified version of a resonant matching network inserted between the driving circuit and the impedance of UGWIDTs. The design goal is to achieve a compromise between the desired bandwidth and power transfer/conversion, also considering the characteristics of the driving electronic circuit while keeping the network as simple as possible. From the following analysis, a design methodology is proposed that allows UGWIDTs to be driven with low-voltage driver circuits (e.g., <24 V), with the advantage of avoiding high-voltage power supply circuits.

Matching networks for UGWIDTs require alternative solutions to those traditionally used for ceramic resonant transducers [11,12]. For the latter, a compensating inductor is used, either in parallel or in series with the transducer. Its electrical impedance is well represented by the resonant circuit model proposed by Kino-Mason in [11]. As is well known, for these transducers, the value of the compensating inductance essentially cancels out the effect of the capacitive component at the resonant frequency (series or parallel) and makes the load impedance equivalent purely resistive. For narrowband systems, matching networks are used to adapt the transducer impedance to the front-end electronics and typically, for single-element transducers, passive networks with L, C and magnetic transformers components are used. The magnetic transformer solution is well known but requires the replication of transformers for the number of elements that constitute the UGWIDT network of the SHM system. In addition to the poor repeatability of the characteristics and the cost, there is an increase in weight and the reliability factor that discourages the adoption of this solution. However, the advantage of the transformer lies in the possibility of increasing the excitation voltage of the piezoelectric transducer starting from driving with low-voltage signals. The matching network design for wide bandwidth piezoelectric transducers is an even more complex network [13] and a recent review of the matching network for ultrasonic transducers is reported in [14]. The use of T- and PI matching networks for piezoelectric transducers was proposed by Chen et al. in [15]. These two types of networks are particularly suitable for solving the impedance matching problem with the connecting cable used for resonant piezoelectric transducers. The solution requires the symmetry of the network with two inductors of equal value, which limits the reproducibility of the network functions, given the inductance value tolerance, which is typically worse than 5% over the entire temperature and current rating range. Furthermore, T- and PI-type networks with three L-C reactive components do not isolate the DC component that may be present in the excitation signal (e.g., a unipolar pulse burst generated by an H-bridge electronic driver). The presence of the DC component represents a significant limitation for the reception phase when the transducer is operated in pulse-echo mode and is coupled to the receiver circuit via a duplexer circuit. For completeness, the two-component reactive matching network was proposed by Moon et al. [16] for impedance matching of resonant ultrasonic transducers for high-frequency applications from 20 MHz to 60 MHz. In [16] the L-C network topology is chosen as a high-pass or low-pass filter depending on the ratio of the source impedance to the transducer impedance.

In the case of UGWIDTs, the classical solutions mentioned above can be adopted, but since the resistive portion of the impedance is generally higher than that of ceramic transducers, it is difficult to transfer electrical power to a high-value resistive load without increasing the excitation voltage to values that require a more complex electronic driving circuit. Furthermore, the ideal matching network should be able to match different values of the output impedance of the driving circuit, which can vary significantly depending on the adopted solution: pulsers with power MOSFET devices or linear amplifiers [17,18]. This aspect has been considered in the choice of matching network topology and is justified by a theoretical analysis of the network functions. This article presents a solution that introduces a resonant matching network with the UGWIDT equivalent impedance parallel to the inductor of the resonant circuit. A matching network design methodology is presented that can be applied to different UGWIDT fabrication technologies based on bandwidth, source impedance and conversion efficiency requirements.

Finally, the presented design solution, based exclusively on a network with two passive components (L and C), will also be evaluated for integrated implementation on the UGWIDT connector to minimize costs, size and weight. The experimental section therefore includes some examples of UGWIDTs made of piezopolymer film (PVDF), since this material is very versatile for building SHM systems but is not as efficient at generating UGWs as piezocomposite materials.

## 2. Matching Network with a Double Tuning Resonant Circuit

**Figure 1:**
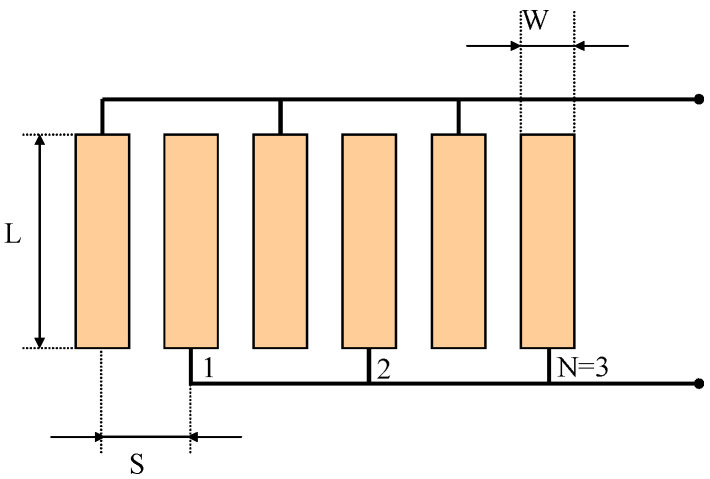
Dimensional parameters of the finger electrodes for a UGWIDT. In the example the number of finger pairs *N* is 3.

For a UGWIDT, it can be assumed that according to the interdigital finger geometry shown in Figure 1, the equivalent electrical capacitance can be estimated by the well-known formula:

(1) $$C_{L}=\varepsilon_{0}\varepsilon_{r}\frac{A}{t}$$

where t is the thickness of the piezoelectric material, A is the active area of the UGWIDT and $\varepsilon_{r}$ is the relative dielectric constant of the piezoelectric evaluated at the operating frequency f and temperature T.

According to Figure 1, the active area A can be calculated as:

A = L x W x N(2)

By applying the KLM model [11] for an IDT, the equivalent resistance $R_{L}$ of the electrical impedance can be estimated to be associated with the conversion of the active electrical power provided to the UGWIDT and the power losses of the material at the given operating temperature T and frequency f. The influence of the piezoelectric material parameters on the equivalent electrical impedance is reflected in the capacitance C_L_ via the relative dielectric constant of the material, as reported in Equation (1), and in the equivalent resistance R_L_. For the latter, its value depends on the acoustic coupling between the piezoelectric material and the material of the monitored structure (most often represented by planar metallic structures made of aluminum or composite materials made of glass or carbon fiber). Providing this information, the equivalent resistance can be estimated using the KLM model (see Castillo et al. [19]). Piezoelectric materials typically used for the construction of UGWIDTs are piezoelectric ceramics and piezoelectric polymers, often in their composite forms. The key parameters are therefore the k_t_ coefficient and the acoustic impedance of the piezoelectric material. For internal power loss, the mechanical loss tangent and the dielectric loss factor must also be considered, both of which influence the equivalent R_L_ value. Furthermore, Wilcox et al. [20] proposed a one-dimensional model for calculating the acoustic impedance of a piezoelectric transducer, with application to a piezopolymer material.

**Figure 2:**
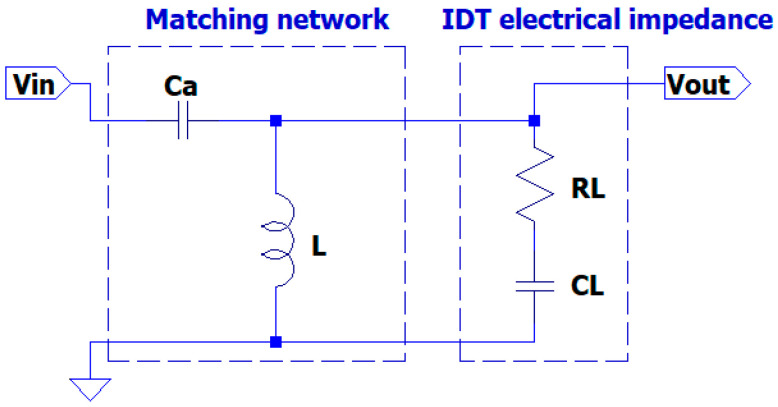
The equivalent transducer impedance Z_L_ connected in parallel to the inductor L.

The matching network circuit proposed in this work is based on a composite resonant L-Ca circuit where the transducer impedance $Z_{L}$ is connected in parallel to the inductor L, as shown in Figure 2. The advantages of this topology will be explained in the following sections, but we can immediately depict it in the figure below.

Notice that the capacitor Ca provides isolation from the eventual DC component in the driving signal Vin and the inductor L is a short circuit for the DC components on Vout. For the completeness of the topic, the solution for a matching network of a capacitive–resistive load to a resistive voltage source can also be performed with two inductors [21] (Ca is replaced by a second inductor), but it is more expensive, bulky and it is a short circuit for the eventual DC component in the driving signal Vin.

The transfer function of the circuit shown in Figure 2 can be solved by MATLAB symbolic equation tools and can be obtained by the following expression in the Laplace domain (s = jω = j2πf):

(3) $$H_{1}\left(s\right)\;=\;\frac{Vout}{Vin}\;=\;\frac{sL\;R_{L}+L/C_{L}}{sL\;R_{L}+\frac{R_{L}}{sC_{a}}+L\;(\frac{C_{L}+C_{a}\;}{C_{L}C_{a}})\;[1+\frac{1}{s^{2}L\;\left(C_{L}+C_{a}\right)}]}$$

The analysis of the denominator of (3) shows a main resonant effect for the angular frequency $\omega_{1}\;=\;\frac{1}{\sqrt{L\left(C_{L}+C_{a}\right)}}$.

The transfer function evaluated for $\omega_{1}$ is simplified to two terms and can be analyzed for new insights relative to the design of the matching network.

(4) $$H_{1}\left(s_{1}=j\omega_{1}\right)=\frac{Vout}{Vin}=\frac{s_{1}\;R_{L}+1/C_{L}}{s_{1}\;R_{L}\;[1+\frac{1}{{s_{1}}^{2}LC_{a}\;}]}=-\left(1+\frac{1}{{s_{1}\;C}_{L}R_{L}}\right)\frac{C_{a}\;}{C_{L}}$$

In (4) we can observe that the first term in brackets depends only on the UGWIDT’s electrical impedance determined by $C_{L}$ and $R_{L}$, and the second term represents a gain factor $G\;=\;\frac{C_{a}\;}{C_{L}}$ that can be adjusted by the choice of $C_{a}$. Once $C_{a}$ is defined, the desired value for $\omega_{1}$ can be tuned by the value of L. The possibility to tune $\;C_{a}$ and L separately is the advantage of the proposed circuit topology for the matching network.

The increase in the gain factor by $C_{a}$ causes a decrease in the input impedance $Z_{in}$ at the input port Vin (see Figure 2), as reported in (5):

(5) $$Z_{in}\left(s\right)\;=\;\frac{Vin}{Iin}\;=\;\frac{sL\;R_{L}+L/C_{L}}{sL\;+\frac{1}{sC_{L}}+R_{L}}+\frac{1}{sC_{a}}$$

**Figure 3:**
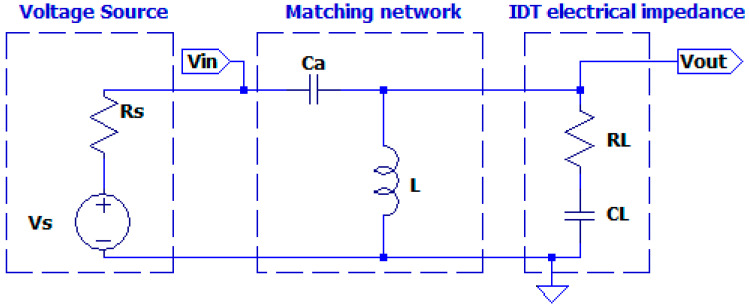
Matching network connected with the input port loading effect of input port Vin to the source Vs and Rs and the electrical impedance of the UGWIDT at the output port V_out_.

In (5) we also observe that the variation of $C_{a}$ and L have independent effects on the two additive terms. Furthermore, we can see from Figure 3 that the effective driving voltage Vin is given by the partition between the source impedance *Zs* and *Zin*. Therefore, a reduction in the value of *Zin* has the effect of decreasing the driving voltage Vout on the transducer.

In (5) the denominator shows a series resonant effects on the impedance with a local maximum at the angular frequency $\omega_{2}=\frac{1}{\sqrt{LC_{L}}}$. The difference between $\omega_{1}$ and $\omega_{2}$ depends on the G; this double resonant effect must be considered for the design of the matching network and exploited to obtain the best trade-off.

**Figure 4:**
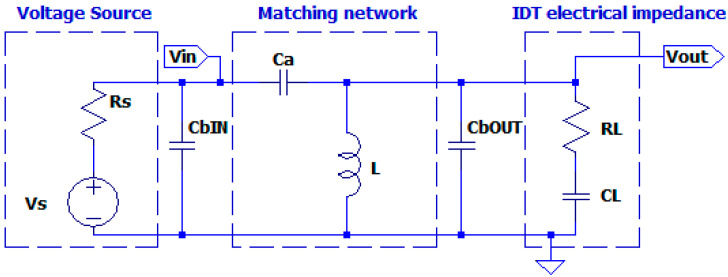
Schematic circuit for considering the capacitive effect of Cb due to the connection cable of the matching network at the input or at the output.

Ideally a matching network is connected directly to the source (driving electronic circuit) with a different output impedance $R_{s}$. In practice the matching network will be connected to the source and the UGWIDT by cables with the line impedance. For the frequency range of interest, we can simplify the effect of the connection line with a capacitor $C_{b}$ in parallel at the input port Vin of the matching network (see Figure 4). We anticipate that this is the circuit that will be treated in detail in Section 2.

For the sake of completeness, we can observe that the series of capacitors Ca and Cb forms a voltage divider for Vout; this circuit network is for matching load at radiofrequency, solving the problem of tuning and matching separately [22]. The higher values of Ca (up to several nF) have an impact on the circuit footprint, volume and cost but the value of the desired resonant frequency is obtained with a lower value of L, which is an advantage especially when currents in the order of 0.1 A–1 A are required for driving the UGWIDT with sufficient power.

### 2.1. Parametric Simulations of the Matching Network

#### 2.1.1. UGWIDT’s Electrical Impedance

**Figure 5:**
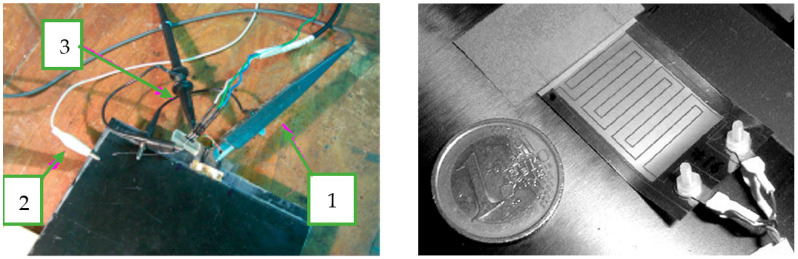
(**Left**) Measurement setup of the equivalent electrical impedance at 450 kHz with volt-amperometric method: (1) current probe P6022 TEKTRONIX, (2) ground reference, (3) voltage probe TEKTRONIX Mod. P3010. The piezopolymer UGWIDT is bonded to the CFRP cross-ply laminate. (**Right**) Piezopolymer UGWIDT.

In this work we consider the design of a UGWIDT with finger electrode pitch S = 4 mm operating at 450 kHz for defect detection in a carbon fiber-reinforced plastic (CFRP) laminate. The electrode design characteristics are reported in Figure 1. The dimensions of the finger electrodes are S = 4 mm; W = 1.5 mm; L = 14 mm; and N = 3. The corresponding central wavelength λ = 2 and S = 8 mm. The piezopolymer film is a gold metallized PVDF with thickness t = 100 µm and the finger electrodes’ geometry is shown in Figure 1. This is realized by laser ablation resulting in the device shown in Figure 5 on the right. The UGWIDT electrical impedance is measured with the UGWIDT mounted on the CFRP cross-ply laminate, as shown in Figure 5.

**Figure 6:**
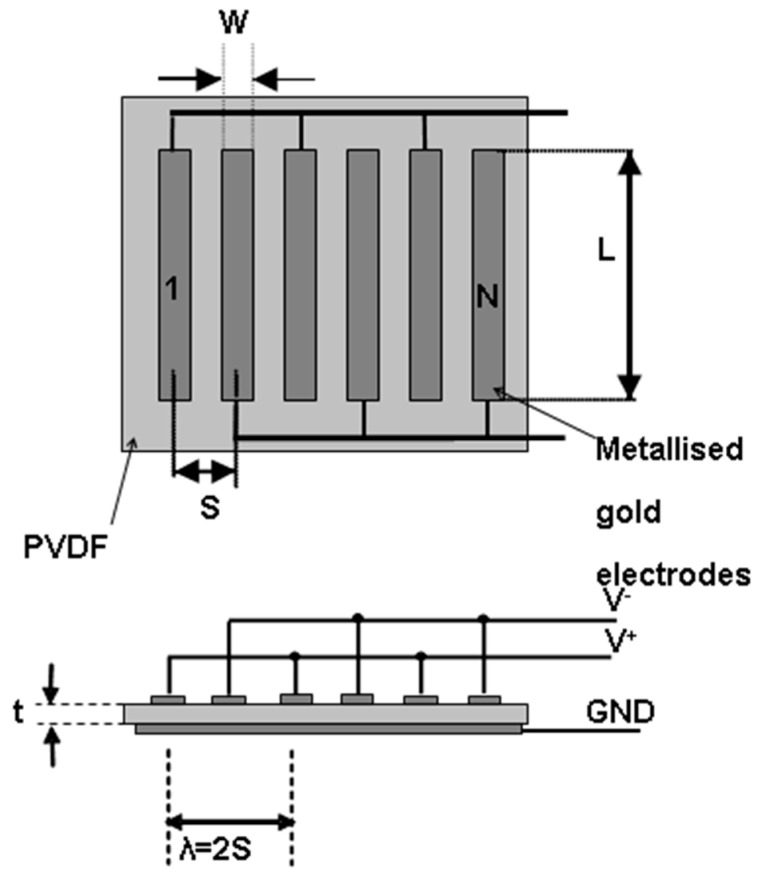
Differential driving V+ and V− of the UWGIDT.

The results for the electrical impedance are a series circuit $C_{L}\;=\;210\;pF,\;R_{L\;}=\;2000\Omega$. This pair of values for $C_{L}$ and ${\;R}_{L}$ corresponds to the electrical impedance of one section of the UGWIDT measured either from V+ or V− to ground (GND), as shown in Figure 6.

In the next section we will use these values for parametric simulations to explain the results of the formal analysis of the transfer functions.

#### 2.1.2. Parametric Simulations for Ca and Rs

The first parametric simulation regards the matching network shown in Figure 3.

The aim is the evaluation of the influence of the choice of the value of Ca on the fixed values of $C_{L}$ and ${\;R}_{L}$.

The value of L = 120 µH is determined by the desired value $\omega_{1}\;=\;2\pi f_{1}$, where $f_{1}\;=\;450\;\mathrm{kHz}$, as reported in Section 2.1. We also consider an inductor series resistance ESR = 0.16 Ω derived from the data sheet of a commercial component. The source resistance Rs = 4 Ω is initially chosen considering a linear power amplifier [23,24]. As previously defined, the value of ω_1 depends on $C_{L}$, $L\;,\;C_{a}$. Therefore, the variation of $\omega_{1}$ due to $C_{a}$ a must be evaluated, with the other two parameters being constant. The analysis of transfer function $H_{1}\left(s\right)$ around 450 kHz will point out this behavior of the $H_{1}\left(s\right)\;$ magnitude according to (4).

**Figure 7:**
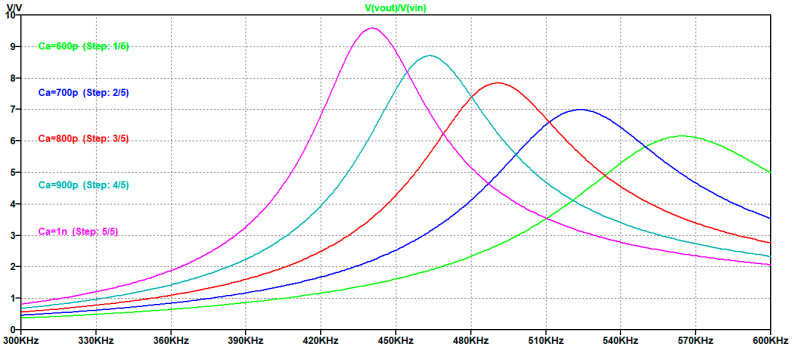
Simulated magnitude of the transfer function $H_{1}\left(s\right)$ with parametric simulations for C_a_. Vertical axis u.o.m is [V/V].

In Figure 7 the simulated magnitude of the transfer function $H_{1}\left(s\right)$ for Ca =600 pF,700 pF,800 pF,900 pF,1000 pF is reported from right to left.

From Figure 7 we observe that the maximum value of the transfer function increases with C_a_ and the evaluation of the magnitude at 450 kHz is reported in the Table 1.

Choosing the higher value of C_a_ equal to 1000 pF provides an amplitude gain factor G = 4.76 and a transfer function amplitude of 8.79. This value of C_a_ is not a limit for the choice, but it is a good compromise for the component’s footprint and parasitic.

Figure 6 also shows the effect of the driving signal that must match the UGWIDT requirements on the bandwidth. Higher values of C_a_ provide narrower bandwidth: for C_a_ = 1000 pF the −3 dB bandwidth is 45 kHz, while for C_a_ = 600 pF it is 72 kHz.

Another important factor for the analysis of the matching network is the effect of variation in the source impedance Rs. Different driver circuits can have a value as low as 0.4 Ω, representing, for example, the case of an MOSFET integrated pulser, much lower than the initially assumed value of 4 Ω for a linear power amplifier. Then a parametric simulation was performed for three values of Rs = [0.4,1, 4] Ω with a fixed value of C_a_ = 1000 pF. The voltage source (see Figure 3) has 15 V amplitude, compatible with integrated driving circuits. The evaluation of the voltage amplitude Vout at the load is reported in Table 2.

The results in Table 2 validate the approach of having a matching network that increases the input voltage from 15 V by a factor 8.7 and is largely independent from the source resistance R_S_.

#### 2.1.3. Capacitive Effect of Connection Cable and Refinement of C_a_

In the theoretical analysis we neglected the effect of the connection cable between the driving electronic circuit and the UGWIDT. In general, this connection for a differential driving of an UGWIDT (see V+ and V− in Figure 6) is realized with a twisted pair shielded cable. Depending on the length and dielectric of the insulation materials, we evaluated the capacitance in parallel to the transducer to be about C_bOUT_ = 170 pF for a 1.2 m length.

**Figure 8:**
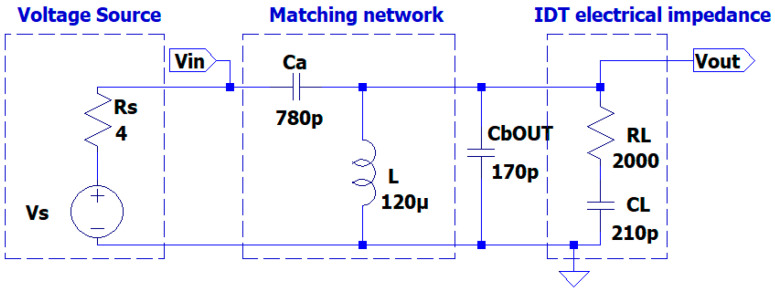
Simulated matching network including connection cable capacitance C_bOUT_ = 170 pF.

Including the cable capacitance in the simulation (see Figure 8), the value of C_a_ must be optimized for the operating frequency of 450 kHz. In the solution proposed in Figure 8, the matching network is located on the board of the electronic circuit and the signals Vout and GND are connected to the transducer by the connection cable. As we will see later in this article, the integration of the matching network on the UGWIDT connector is more convenient because the effect of cable capacitance is reduced.

For the optimization, we select by parametric simulation an optimal value of C_a_ = 780 pF, which maintains a high output voltage Vout on the transducer of 105 V. Accounting for this effect, the transfer function magnitude is 105/15 = 7, lower than 8.79, as evaluated in Table 1, without considering the cable capacitance.

### 2.2. Experimental Validation of the Matching Network

The designed matching network has been mounted onto the PCB of the driving circuit that is a power linear amplifier [25] and connected with a shielded cable to the UGWIDT described in the previous section.

**Figure 9:**
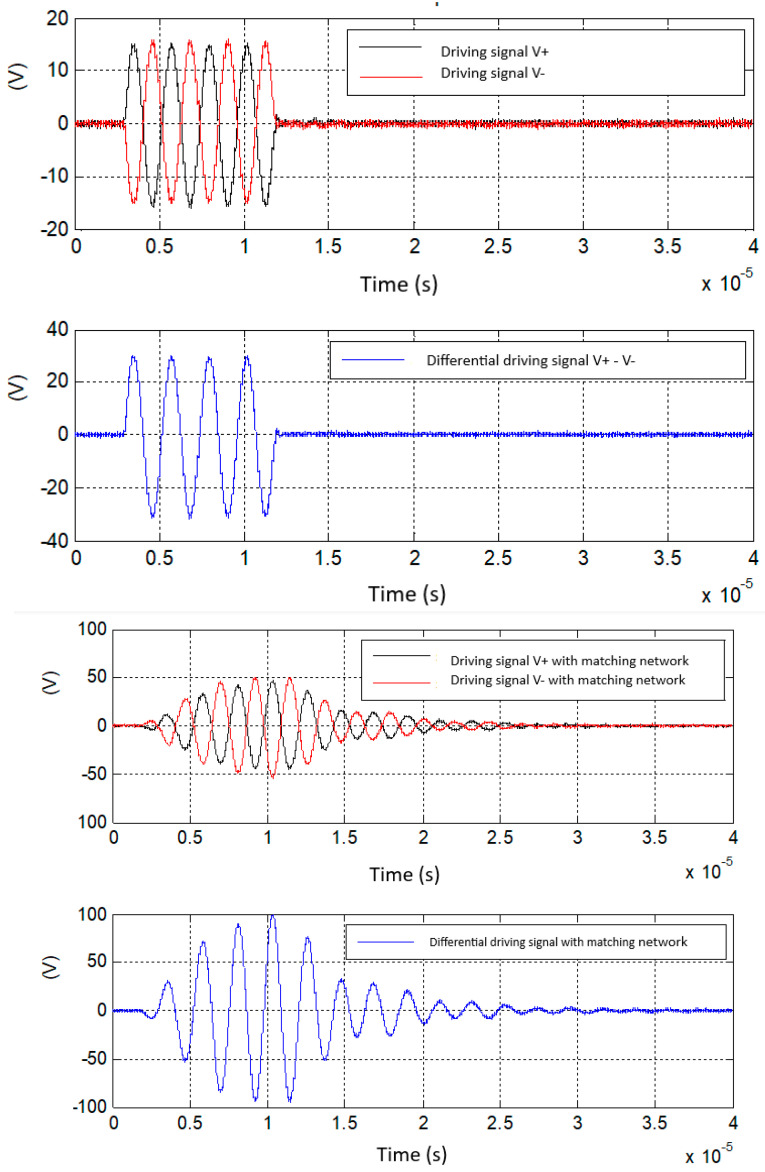
(**Top**) driving signals from a linear power amplifier. (**Bottom**) Driving signals with the matching network described in Figure 8.

The driving differential signals (V+ and V−) applied to each section of the UGWIDT (see Figure 6) are shown in Figure 9 (top).

The experimental results in Figure 9 (top) show driving signals with an amplitude of 15.5 V and in Figure 9 (bottom) the corresponding signals after the insertion of the matching network have an amplitude of about 100 V. The transfer function magnitude experimentally evaluated is 100/15.5 = 6.45. The estimate is in good agreement with simulations considering that some parasitics of passive components are not considered in the simulations.

### 2.3. Evaluation of the Input Impedance $Z_{in}$

The last part of the analysis concerns the estimation of the input impedance at the input port of the matching network.

**Figure 10:**
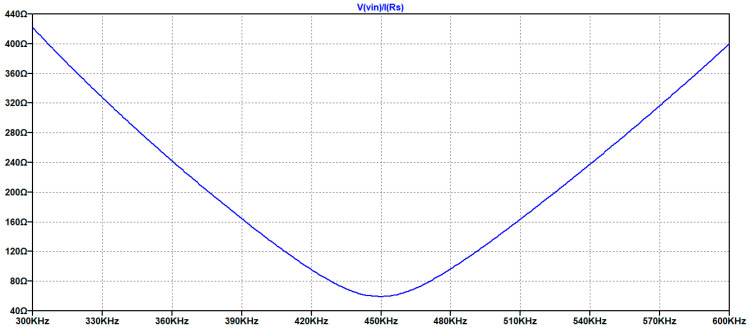
Magnitude of the input impedance Zin with the matching network.

In Figure 10 the magnitude of the impedance has been simulated for the circuit shown in Figure 8, and the minimum is obtained at 450 kHz and its value about 59 Ω.

The minimum magnitude for Zin = 59 Ω confirms the low influence of the source impedance that is, in general, much lower than this value. Then this design approach is versatile for different driving electronic circuits with output impedance lower than this value.

## 3. Integration of the Matching Network on the UGWIDT Connector

In Section 2.1.2 the effect of the cable used for the connection between the matching network and the UGWIDT is pointed out. An alternative approach is the realization of the matching network on a PCB that is also used to connect metal fingers to the driving signals. The type of contact between top and bottom PCBs with copper pads and the gold metallized PVDF is guaranteed by the pressure generated by microrivets joining the two PCBs and the PVDF in the middle [3]. On one of the two PCBs the circuit of the matching network is mounted with low profile and low weight SMD components.

**Figure 11:**
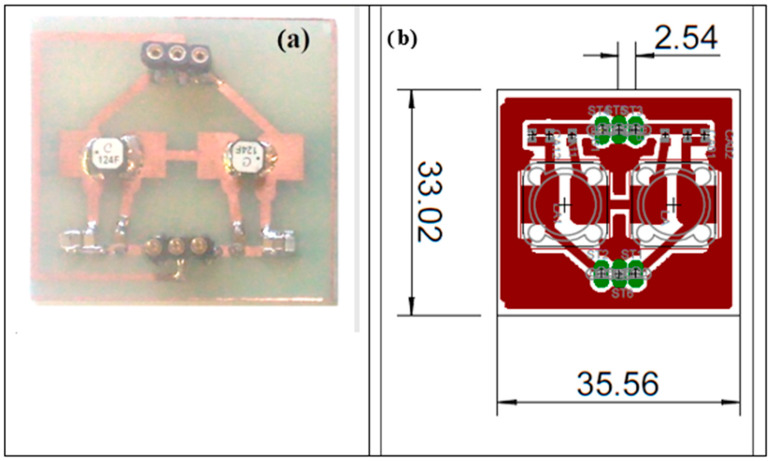
(**a**) Final assembly of the matching network with differential output and ground. (**b**) PCB design for the connector with integrated matching network; dimensions are in mm.

In Figure 11 the PCB design and the mounted matching network are illustrated.

**Figure 12:**
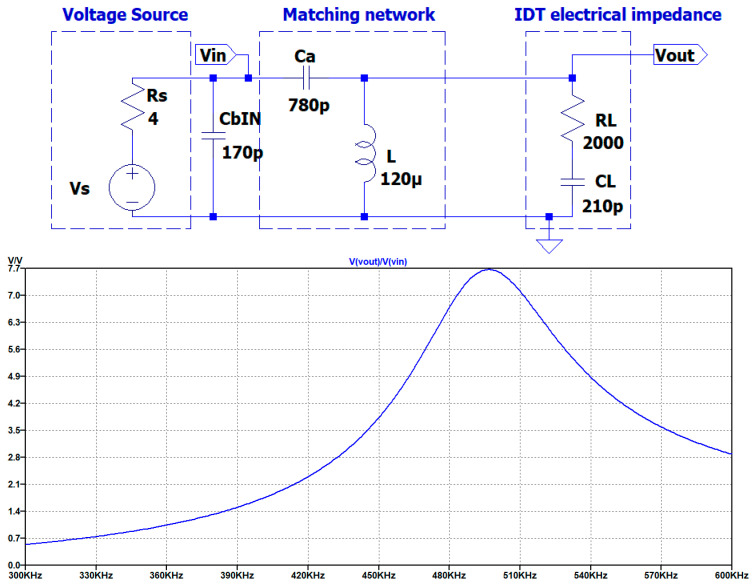
(**Top**) Schematic of the matching network including the connection cable capacitance C_bIN_ = 170 pF to the driving electronics. (**Bottom**) Simulated magnitude of the transfer function. Vertical axis u.o.m. [V/V].

With this change in the connection, now the cable capacitance must be moved in parallel to the input of the matching network, as shown in Figure 12.

In the bottom of Figure 12 we can see that at 450 kHz the gain factor evaluated on the magnitude of $H_{1}\left(s\right)$ is 3.8. By simulation we can also observe a change in the Z_in_ that is increased to 91Ω at 450 kHz while the minimum equal to 51 Ω is shifted to 494 kHz. The higher value of the input impedance means the requirement of higher input driving voltage to increase the current in R_L_. This problem will be tackled in the next section.

## 4. Analysis of the Matching Network Implementation Considering the Experimental Environment

The operating environment is certainly relevant to the implementation of the matching circuit, especially in avionics and space applications. For this reason, the first analysis was conducted considering the implementation of the network on the driver circuit board and, consequently, the effect of the capacitance of the connecting cable to the UGWIDT; in this case, environmental variables are mitigated by the choice of a robust electronics enclosure and by temperature conditioning. In the second case, the matching network, being integrated with the transducer, is exposed, like the transducer itself, to environmental variables. In this situation, the piezoelectric transducer is the most sensitive element of the system. For example, the choice of a piezopolymer material is advantageous in the presence of significant mechanical shocks, vibrations and structural expansions greater than 5% due to temperature variations, since this material is not as brittle as, for example, piezoceramic. The effects of temperature on the variation in piezopolymer material parameters were initially studied by Ohigashi [26] and Omote et al. [27].

Regarding the choice of reactive L-C components, we can refer to the datasheets of the SMD components used to create the networks proposed in this article. Specifically, the Coilcraft LPS4018 SMD inductor features are as follows:

- High performance with a compact profile;
- High energy storage and low DC resistance;
- Magnetic shielding allows for high-density mounting;
- AEC-Q200 Grade 1 (−40 °C to + 125 °C);
- Tolerance = 20%.

Note that the inductor’s temperature range is wider than that of piezopolymer materials, which typically do not exceed 80 °C. Conversely, there are no strict environmental constraints for capacitor selection.

Finally, the proposed LC matching network can be integrated with the transducer PCB and protected from the external environment by encapsulation.

## 5. Pulser Circuit with Matching Network

In the previous section we concluded that the matching network can be designed with adequate gain factor and bandwidth, but the high input impedance limits the power delivered to R_L_. For this inconvenience, a driving circuit based on an H-bridge with four power MOSFETs is an effective solution. This type of electronic circuit is now also common in integrated devices and can be supplied with a single power supply voltage [28,29,30,31]. The output voltage is doubled across the load thanks to the differential connection.

**Figure 13:**
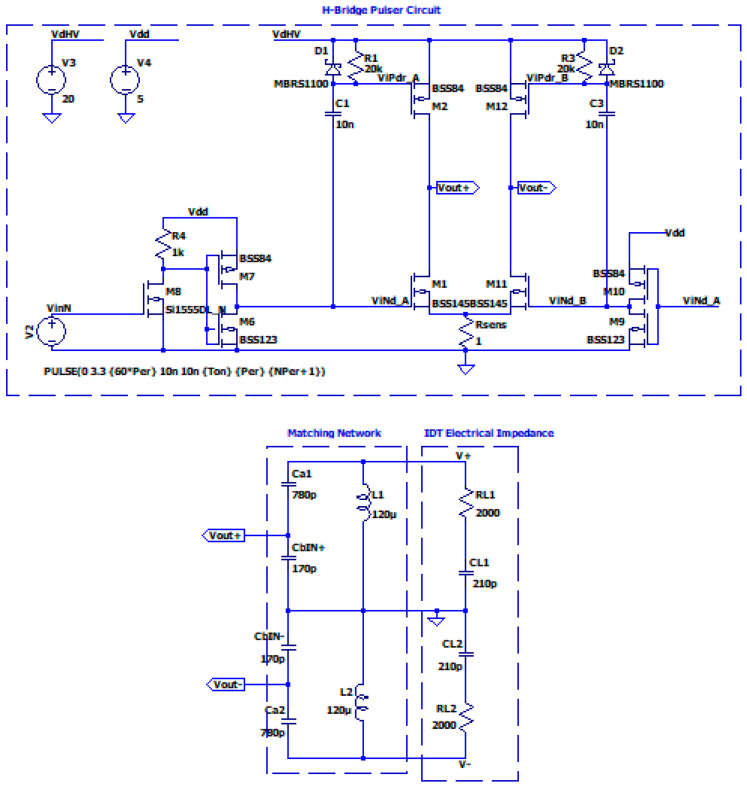
Simulation of an H-bridge pulser circuit connected to the matching network with a cable capacitance of 170 pF. Power supplies are +20 V for the driver and +5 V for the digital input interfaces with an externally generated burst of unipolar pulses.

In a previous project we developed a modular system for an exciting array of 16 IDTs [32]. Here we use the same board to test the effectiveness of the matching network for UGWIDTs. In Figure 13 the schematic circuit for the simulation is shown.

The excitation signal is a burst of four pulses at 450 kHz at 5 V. These pulses are then processed to get out of phase-correspondent signal by a CMOS inverter (see bottom-right of Figure 13). The driving signals (Vleg+ and Vleg−) are obtained with a power supply voltage Vdd = 20 V. The output signals at the UGWIDT are Vout+ and Vout−.

**Figure 14:**
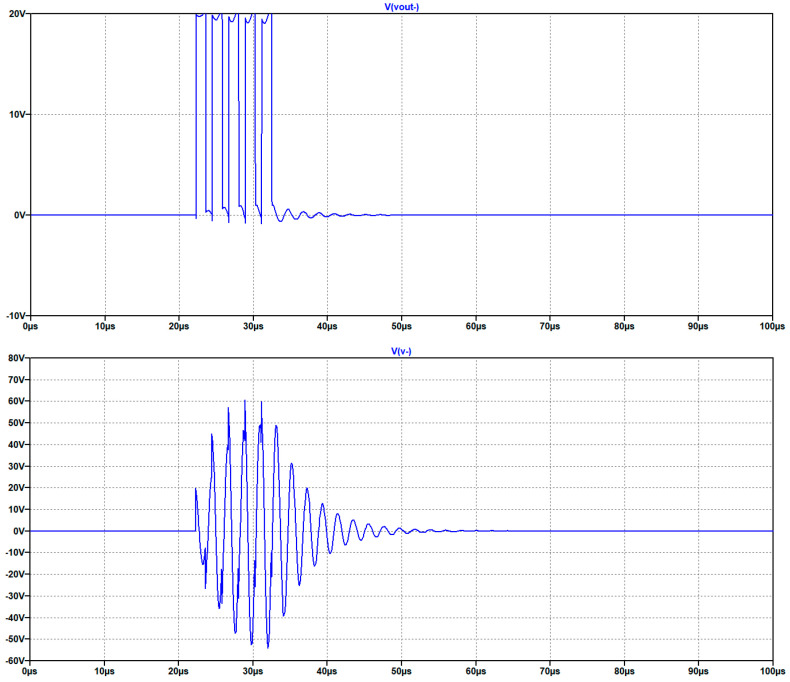
Simulation results of the excitation with an H-bridge supplied at 20 V. (**top**) Driving signal for one section of the H-bridge. (**bottom**) Output voltage V+ applied to one section of the UGWIDT.

In Figure 14 the results of the simulations are illustrated.

Through simulation we can observe that a burst with an amplitude of 20 V generates a driving signal at one section of the UGWIDT of about 54 V. The evaluation of the magnitude is 54/20 = 2.7. The peak to peak is about 108 V. The pulser circuit with differential driving recovers from the limitation of the integrated linear power amplifiers that can drive the load at the maximum current up to 24 V.

The simulated results also need to be validated with an electronic circuit board implementing the H-bridge circuit shown in Figure 13 and the matching network realized on a PCB for connecting the piezopolymer UGWIDT.

**Figure 15:**
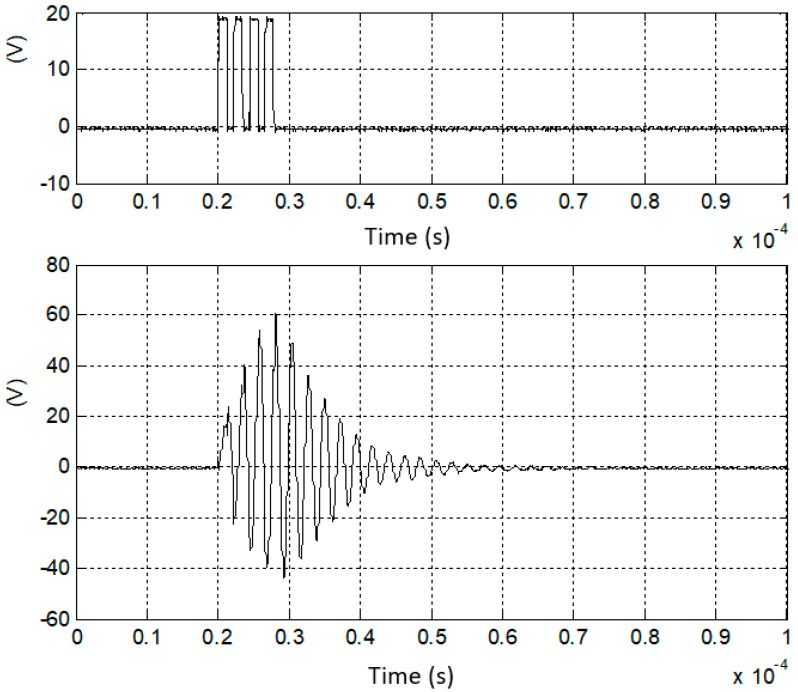
(**Top**) Excitation burst with four unipolar pulses applied to one input (pin Vout−) of the differential matching network shown in Figure 13. (**Bottom**) Output signal (see pin V− in Figure 13) applied to one section of the UGWIDT.

In Figure 15 the excitation signals with four pulses at 450 kHz with 20 V amplitude are presented along with the corresponding signal on one section of the UGWIDT.

The comparison of the simulations and the experimental results shows that the gain factor and the shape of the output signal are in good agreement, and this solution can be adopted efficiently for driving the input impedance of the matching network.

## 6. Conclusions

The design of the matching networks of UGWIDTs fabricated with piezopolymer films is presented. Because of their relatively high impedance at the operating frequencies for SHM systems and the low amplitude of the driving signals generated by integrated electronics, a matching network based on resonant behavior is proposed and analyzed for the specific user case. The theoretical analysis of the transfer function and the input impedance provides insight into how to select the two main components, L and Ca, of this matching network with the load inserted parallel to the inductor. In this way we can separately design the value of L for the desired operating frequency and the value of Ca for the voltage amplitude gain factor. The matching network is simple and provides a relatively high impedance with respect to the source, minimizing the effect of performance change depending on the driving electronics. Finally, the solution with an integrated matching network on the UGWIDT connector is presented and the full experimentation with an H-bridge pulser demonstrates the effectiveness of this simple solution. The driving voltage up to 100 Vpp can be achieved by increasing the signals generated from integrated power amplifiers or pulsers, typically in the order of 24 V. The design rules and the main trade-offs are reported so that the design of this type of matching network can be extended for UGWIDTs fabricated with other piezoelectric materials.

## Figures and Tables

**Table 1:** Magnitude of $H_{1}\left(s\right)$ and factor G evaluated at f = 450 kHz for different values of C_a_.

C_a_	$\left|\;H_{1}\left(s\right)\;\right|$	G
600 pF	1.62	2.85
700 pF	2.57	3.33
800 pF	4.27	3.80
900 pF	7.6	4.20
1000 pF	8.79	4.76

**Table 2:** Evaluation of Vout at frequency of 450 kHz for different values of Rs.

Rs [Ω]	Vout [Volt]
0.4	133.2
1	129.6
4	121.5

## Data Availability

No new data were created or analyzed in this study. Data sharing is not applicable to this article.

## Abbreviations

The following abbreviations are used in this manuscript:

UGWIDT	Ultrasonic Guided Wave Interdigital Transducer
SHM	Structural Health Monitoring
IDT	Interdigital Transducer
PCB	Printed Circuit Board
PVDF	Polyvinylidene Difluoride
